# Rare Gene Mutations in Romanian Hypoacusis Patients: Case Series and a Review of the Literature

**DOI:** 10.3390/medicina58091252

**Published:** 2022-09-09

**Authors:** Alexandra-Cristina Neagu, Magdalena Budișteanu, Dan-Cristian Gheorghe, Adela-Ioana Mocanu, Horia Mocanu

**Affiliations:** 1 Department of ENT&HNS, “Marie Sklodowska Curie” Emergency Children’s Hospital, 041434 Bucharest, Romania; 2 Department of Medical Genetics, Faculty of Medicine, “Titu Maiorescu” University, 031593 Bucharest, Romania; 3 Department of ENT&HNS, Faculty of Medicine, “Carol Davila” University of Medicine and Pharmacy, 020021 Bucharest, Romania; 4 Department of ENT&HNS, Polimed Medical Center, 040067 Bucharest, Romania; 5 Department of ENT&HNS, Faculty of Medicine, “Titu Maiorescu” University, 031593 Bucharest, Romania

**Keywords:** genetics of hearing loss, mutations, rare, hypoacusis, mitochondrial, TWNK, PACS2, SYT2, SUCLG1, genotype–phenotype correlation

## Abstract

(1) Background: In this paper, we report on three cases of hypoacusis as part of a complex phenotype and some rare gene variants. An extensive review of literature completes the newly reported clinical and genetic information. (2) Methods: The cases range from 2- to 11-year-old boys, all with a complex clinical picture and hearing impairment. In all cases, whole exome sequencing (WES) was performed, in the first case in association with mitochondrial DNA study. (3) Results: The detected variants were: two heterozygous variants in the TWNK gene, one likely pathogenic and another of uncertain clinical significance (autosomal recessive mitochondrial DNA depletion syndrome type 7—hepatocerebral type); heterozygous variants of uncertain significance PACS2 and SYT2 genes (autosomal dominant early infantile epileptic encephalopathy) and a homozygous variant of uncertain significance in SUCLG1 gene (mitochondrial DNA depletion syndrome 9). Some of these genes have never been previously reported as associated with hearing problems. (4) Conclusions: Our cases bring new insights into some rare genetic syndromes. Although the role of TWNK gene in hearing impairment is clear and accordingly reflected in published literature as well as in the present article, for the presented gene variants, a correlation to hearing problems could not yet be established and requires more scientific data. We consider that further studies are necessary for a better understanding of the role of these variants.

## 1. Introduction

Hearing loss of variable etiology represents a serious health issue worldwide with over 250 million people currently afflicted (data reported by the World Health Organization), which represents 4.2% of the world’s population [1,2,3]. Congenital hearing loss is also relatively frequent, with a prevalence reported by different sources in literature as varying between 1–3/1000 newborns [4] and 1/500 newborns [5].

Although the etiology includes various factors, at least 50% of all early onset hearing losses have a genetic cause, and of these, the large majority, 75–80%, are most probably autosomal recessive; 70% are non-syndromic. The rest of the congenital hearing losses are determined by clinical and environmental factors such as ototoxic medication, prematurity, or complications at birth. [6].

Due to the etiological heterogeneity of congenital hearing loss, genetic, clinical, and environmental risk factors often combine to provide a complex picture that makes genetic evaluation and council extremely difficult, especially for very small children [6].

Mutations in genes related to mitochondrial functions cause numerous disorders that affect the nervous system and are sometimes associated with congenital hypoacusis and ophthalmic neuropathy. We should also note that many neurological conditions are caused by immensely heterogenous gene mutations, which often require complex diagnosis [7].

Mitochondrial diseases are disorders caused by impairments of the mitochondrial respiratory chain. The genetic error can affect both mitochondrial DNA (mtDNA) and nuclear DNA (nDNA) [8,9]. Human mtDNA is a double-stranded, circular molecule encoding 13 protein subunits, 2 ribosomal RNAs (rRNAs), and 22 transfer RNAs (tRNAs) [10,11]. Visual and auditory pathways, heart, central nervous system (CNS), and skeletal muscle are the tissues mostly involved, because of their dependence on aerobic energy production [8]. Congenital hypoacusis is also common in patients with mitochondrial disorders, eventually affecting over half of all cases in the course of the disease [10]. The clinical presentation of mitochondrial deafness varies considerably, both in terms of associated clinical features and of natural history. In some patients, deafness is only part of a multisystem disorder, often involving the central nervous system, neuromuscular system, or endocrine organs; in other cases, deafness may represent a feature of an oligosyndromic disease [12]. There are also a number of mitochondrial “pure” deafness disorders, most of which probably maternally inherited, most frequently due to the A1555G mutation in the 12 s rRNA gene (m.1555A > G in MTRNR1, also known as 12SrRNA) [13] and m.3243A > G in MTTL1 (tRNALeu(UUR)) [14]. We should also note that the A1555G mutation is associated with aminoglycoside ototoxicity and nonsyndromic SNHL, while MTTL1 mutation is associated with mitochondrial encephalomyopathy, lactic acidosis, and stroke-like episodes (MELAS); maternally inherited diabetes and deafness syndrome (MIDD); and chronic progressive external ophthalmoplegia (CPEO) [11]. A number of other mitochondrial mutations, such as 961delT/insC, T1095C, C1494T, A1555G, and possibly A827G, T1005C, and A1116G in MTRNR1, and G7444A, m.7445A> G, 7472insC, and 7511T > C in MTTS1 (tRNASer(UCN)) have been associated with nonsyndromic SNHL [14,15,16,17,18,19].

The clinical features of mitochondrial hearing loss are: maternal inheritance and sensorineural and primarily symmetrical hearing loss, with involvement of higher or all frequencies; variable penetrance and severity; and in general, childhood onset (postlingually) [14].

In this paper, we report on three cases of Romanian children with sensorineural hearing loss in association with a complex phenotype, each with a different, rare gene variant.

## 2. Case Presentation

### 2.1. Case 1

The first case is a 2-year-old male patient born full term via C-section after an uncomplicated pregnancy, with a birth weight of 3800 g, an Apgar score of 9, and good postnatal adaptation. Both parents were healthy and denied family medical history or consanguinity. In the first ten months of life, he had normal psychomotor development. Around 11 months of age, parents noted that he was struggling to crawl and was not as active, seemed to have low tone, and lost his balance frequently. He also appeared more agitated and nervous. The patient presented to the Marie Sklodowska Curie Emergency Children’s Hospital on 2 October 2020, where he was extensively evaluated by a multidisciplinary team, including ophthalmology, ENT with complete audiological testing (auditory brainstem response—ABR, behavioral observation audiometry, middle immittance audiometry, Distortion Product Otoacoustic Emissions—DPOAEs), and neurology, EEG, brain MRI, skin biopsy, Fluoroscopy Swallowing Function Test, Sleep Study and Whole exome sequencing (Centoxome GOLD^®^ and CentoMito^®^ Genome, CENTOGENE AG, Rostock, Germany).

**Figure 1:**
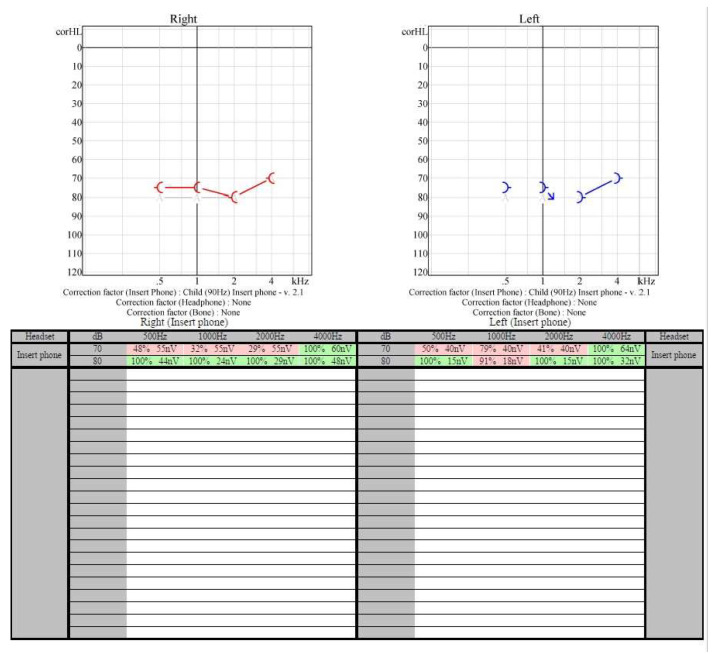
Audiometry Steady State Response results for Case 1 (Severe bilateral sensorineural hearing loss).

**Figure 2:**
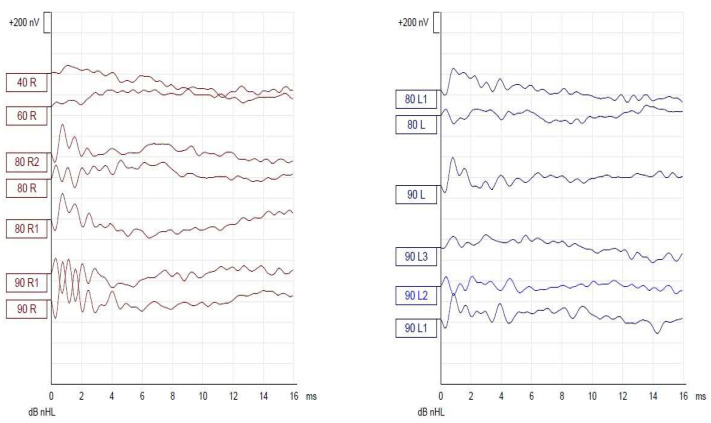
Auditory Evoked Potentials results for Case 1. Cochlear microphonic potentials to be observed bilaterally.

**Figure 3:**
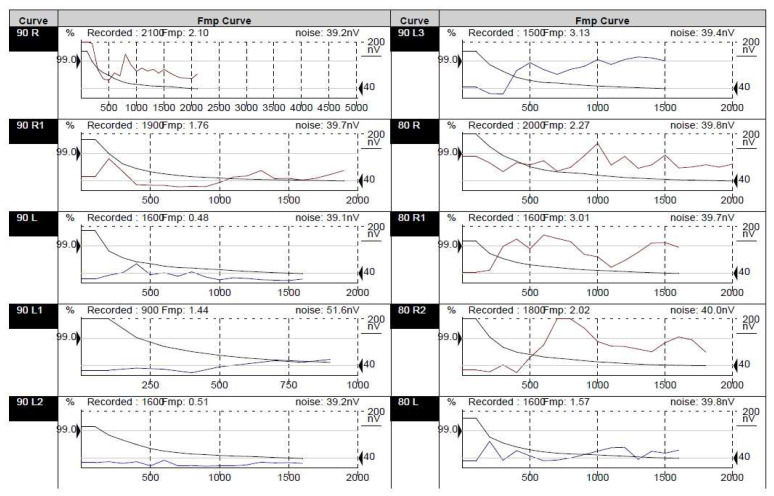
Auditory Evoked Potentials results for Case 1 (extended results). No evoked response until 90 dB nHL bilaterally.

EEG and MRI were normal. The eye exam noted evidence of optic atrophy and neuropathy of the left eye. Audiological evaluation was apparently normal initially (otoacoustic emissions screen passed at the age of 1). One year later, a more extensive examination that included hearing assessment in the sound field for specific frequencies and speech using behavioral observation audiometry, Auditory Steady State Response Audiometry (Figure 1), auditory brainstem response (ABR) evaluation (Figure 2 and Figure 3), middle ear function using immittance audiometry and cochlear outer hair cell function using otoacoustic emissions. Behavioral test results were obtained with fair reliability for the speech stimuli. Responses to speech stimuli were obtained at 70 dB HL in the sound field condition. No reliable responses to frequency-specific stimuli in the sound field could be obtained; however, no noticeable behavioral responses to stimuli at the limits of the equipment were observed. Tympanometry revealed type A tympanograms bilaterally. Ipsilateral acoustic reflexes were absent at 1000 Hz bilaterally. Distortion Product Otoacoustic Emissions (DPOAEs) were present from 1500–8000 Hz bilaterally. ABR indicated auditory neuropathy. This concludes that hearing sensitivity was in the severe hearing loss range in the sound field to speech stimuli. No reliable responses to frequency-specific stimuli could be obtained. Immittance audiometry findings showed normal middle ear pressure and mobility. Acoustic reflexes were absent bilaterally. Otoacoustic emissions suggest normal cochlear outer hair cell function bilaterally.

The patient developed constant abnormal movements involving his upper extremities. He also had significant challenges with feeding and weight gain. A variety of tests, including liver function test, cholesterol profile, and other biological tests, was completed, which were normal. A few episodes of choking on food were also documented. A sleep study showed evidence of obstructive sleep apnea. A skin biopsy was also completed as a part of Mito EpiGemonics Study. Fluoroscopy Swallowing Function showed no evidence of aspiration or penetration, such as +poor nutritive suck with Dr Brown level 3 nipple, prolonged transit with pureed, vertical jaw movements with the mastication of solids, or sometimes swallowing pieces whole.

Whole exome sequencing (Centoxome GOLD^®^ and CentoMito^®^ Genome, CENTOGENE AG, Rostock, Germany) showed compound heterozygous variants in TWNK gene (c.1000C > T/ p.Arg334*—maternally inherited and c.1358G > A/p.Arg453Gln—paternally inherited) and heterozygous variant in SPTBN2 (c.6881A > G/ p.His2294Arg).

Cochlear implantation was discussed, but, unfortunately, due to the complex neurological problems, repeated episodes of aspiration, chocking and pneumonia, the child died at the age of two and a half.

### 2.2. Case 2

The second case is a 4-year-old male patient born from non-consanguineous healthy parents, after a normal twin pregnancy (his twin sister has no health issues), at 34 weeks with a birth weight of 1860 g and an Apgar score of 7. Initial postnatal adaptation was good, but on the fourth day of life, an acute pulmonary hemorrhage occurred, with NICU admittance and intubation for 4 days with a poor subsequent postnatal adaptation. He has an older brother with mild hearing impairment of undetermined etiology without other health issues. The child had a history of left focal epileptic seizures with the onset at the age of 3 months for which he was evaluated in the department of Pediatric Neurology. Multiple EEG examinations as well as 2 brain MRI scans within 7 months of each other, were performed (the first showed normal results, the second showed signal modifications in the mesencephalon and the posterior-medial region of the putamen). At the age of 1 year, on 23 January 2019, he was referred to the ENT department for a suspicion of hearing impairment. At the age of 4 years, the clinical phenotype included growth delay (weight of 12 kg and height of 92 cm), a dysmorphic facial feature (narrow bitemporal diameter, hypertelorism, epicanthus, and low-set ears), high-arched palate, dental anomalies, abnormality of skin pigmentation, talipes equinovarus, strabismus, microcephaly, muscle hypotonia, and global developmental delay. Other examinations included heart and abdominal ultrasounds, EMG exam and ophthalmological examination, all with normal results.

**Figure 4:**
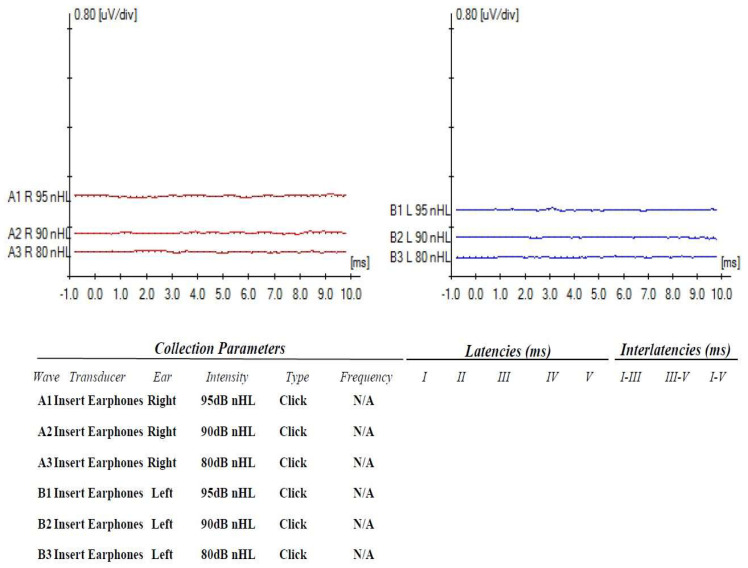
Auditory Evoked Potentials results for Case 2. No evoked response upon maximal stimulation (95 dB nHL) bilaterally.

**Figure 5:**
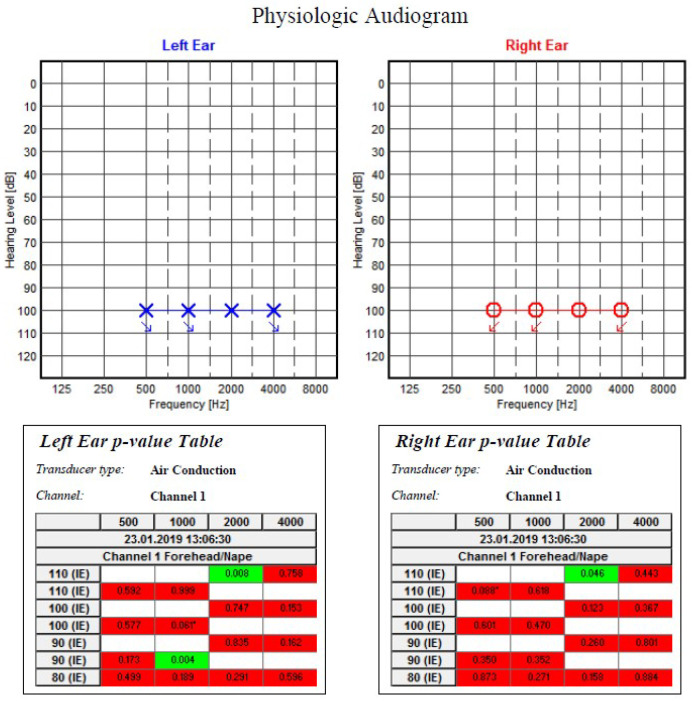
ASSR results for Case 2 (Profound bilateral sensorineural hearing loss).

For establishing the diagnosis of profound bilateral sensorineural hearing loss, Auditory Evoked Potentials (Figure 4) and Auditory Steady State Response Tests (Figure 5) as well as DPOAEs (absent bilaterally from 1500 to 8000 Hz) and a Tympanogram were performed. Immittance audiometry findings showed normal middle ear pressure and mobility. Acoustic reflexes were absent bilaterally. Cochlear implantation of the left ear was performed in August 2019 (at the age of 1) with good functional results.

Whole Exome Sequencing (Centoxome GOLD^®^, CENTOGENE AG, Rostock, Germany) was also performed. WES identified a heterozygous variant of uncertain significance in the PACS2 and SYT2 gene.

The PACS2 variant c.1792G > A p.(Val598Met) causes an amino acid change from Val to Met at position 598. This variant has been confirmed by Sanger sequencing. It is classified as variant of uncertain significance (class 3) according to the recommendations of Centogene and ACMG.

The SYT2 variant c.326A > T p.(Lys109Met) causes an amino acid change from Lys to Met at position 109. It is classified as variant of uncertain significance (class 3) according to the recommendations of Centogene and ACMG.

### 2.3. Case 3

The third case is a 11-year-old male patient who was referred to the ENT department on 10 July 2019, at the age of 2 for a suspicion of hearing problems. He was born from non-consanguineous healthy parents, after a normal pregnancy, at 36 weeks of gestation, with a birth weight of 3200 g and an Apgar score of 9 and a good postnatal adaptation. The boy had a history of psychomotor regression and dystonic movements for which he was evaluated in the department of Pediatric Neurology; a brain MRI showed diffuse cerebral atrophy. A psychomotor evaluation at the age of 2 years showed a global developmental delay with a mental age of about 7 months: 12 months for receptive language, 6 months for expressive language, 5 months for motor development, and 3 months for affective behavior.

For the diagnosis of profound bilateral sensorineural hearing loss, Distortion Product Acoustic Otoemissions (no distortion product between 750—8000 Hz), Brainstem Evoked Response Audiometry (evoked auditory response absent bilaterally at maximum intensity of 90 dB nHL) and Auditory Steady State Response Tests (no evoked auditory answer at 100 dB nHL on all tested frequencies—500, 1000, 2000 and 4000 Hz) as well as a Tympanogram (normal middle ear pressure and mobility, acoustic reflexes present bilaterally only at 46 Hz and 100 dB) were performed. Cochlear implantation was performed in 2014 (at the age of 3 years and 10 months) with good functional results.

Whole Exome Sequencing (Centoxome PLATINUM^®^, CENTOGENE AG, Rostock, Germany) was also performed. WES identified a homozygous variant of uncertain significance in the SUCLG1 gene. The genetic diagnosis of autosomal recessive mitochondrial DNA depletion syndrome type 9 (encephalomyopathic type with methylmalonic aciduria) is considered possible.

## 3. Discussion

All cases presented complex phenotypes including, besides neurological features, sensorineural hearing loss and rare pathogenic mitochondria DNA mutations.

In the first case, WES, in association with mitochondrial DNA study identified two heterozygous variants in the TWNK gene, one likely pathogenic and another of uncertain clinical significance. TWNK gene is mapped on chromosome 10q24 and encodes twinkle and twinky proteins, a mitochondrial protein which has an important role in hexamer formation and DNA bindin [20,21]. Variants of TWNK gene have been associated with Mitochondrial DNA Depletion Syndrome 7 (Hepatocerebral Type, MIM 271245), which is a rare genetic syndrome characterized by hearing loss and neurological features, including ataxia, hypotonia, epileptic seizures, sensory axonal neuropathy, and ophthalmoplegia [22,23]. In most cases the patients develop over time severe refractory epilepsy, with status epilepticus or epilepsia partialis continua, finally evolving into an epileptic encephalopathy [24]; in some cases, death due to refractory epilepsy was reported [24]. No evident phenotype-genotype correlation was reported, thus the reports on individual cases could bring important information for the clinical impact of a specific gene variant.

The phenotype of our patient included most of the features previously reported, including global developmental delay, hypotonia, growth delay, dyskinesia, hearing impairment, optic neuropathy, and polyneuropathy. The absence of epileptic seizures (a main clinical features of the syndrome) in our case could be due to the young age of the patient (14 months). Thus, we consider that the genetic variants of TWNK gene identified in this case are responsible for the clinical features of the patient, including hearing loss.

In the second case heterozygous variants of uncertain significance were identified in two different genes: PACS2 and SYT2. Although known to be associated to congenital hearing loss, SYT2 mutations are rarely reported in literature as such. Hermann et al. report on ten patients with an Autosomal-Dominant Form of Lambert-Eaton Myasthenic Syndrome caused by SYT2 mutations of which only oldest two showed mild hypoacusis [25].

Mutations in PACS2 gene were reported in association with autosomal dominant early infantile epileptic encephalopathy, a clinical feature presented also in our patient.

PACS2 (phosphofurin acidic cluster sorting protein 2) gene encodes a protein with role in nuclear gene expression and ion channel trafficking [26,27].

Mutations of this gene were reported in association with developmental and epileptic encephalopathy 66 (DEE66, MIM 618067), which is a very rare condition with only 14 cases reported to date [27]. The disease is characterized by early-onset epileptic seizures, usually resistant to anti-seizure drugs, global developmental delay, muscle hypotonia, behavioural problems (autism, obssessive-compulsive disorder), ophthalmological problems (astigmatism, myopia or hypermetropia, nystagmus, cortical visual impairment), dysmorphic facial features (hypertelorism, downslanting palpebral fissures, synophrys, broad nasal root, thin upper lip, wide mouth with downturned corners), and other different medical problems (limb malformations, cryptorchidism, cardiac septal defects, anemia, neutropenia, cerebellar anomalies on MRI) [27]. Our patient presented a clinical picture which included many of these features (epileptic seizures, global developmental delay, microcephaly, hypotonia). In that concern the sensorineural hearing impairment, there is only one patient reported by now associating a mild conductive hearing loss [27] in a patient with the variant p.Glu209Lys, different from the variant of our case (p.(Val598Met)), so we cannot make a definite association between this syndrome and the hearing problems but we recommend further research of this matter.

In the third patient a homozygous variant of uncertain significance in SUCLG1 gene was identified. The SUCLG1 (succinate-CoA ligase, alpha subunit) gene encodes the alpha subbunit of mitochondrial succinyl CoA synthetase [28], with role in Krebs cycle [29]. Variants of this gene are associated with mitochondrial DNA depletion syndrome-9 (Encephalomyopathic Type with Methylmalonic Aciduria, MTDPS9, MIM 245400), a rare autosomal recessive disease with a severe phenotype including severe global developmental delay with progressive neurologic deterioration, hypotonia, lactic acidosis and excretion of methylmalonic acid; the onset of the manifestations is usually very early, from the first day of life, with a rapid fatal evolution [29]. There are several reports on cases with the onset of the symptoms after the first 2–3 months of life, but with a rapid progressive neurologic deterioration, with severe hypotonia, severe psychomotor delay, deafness, severe respiratory problems, and lactic acidosis, with fatal evolution in the first 2–3 years of life or with survival till the age of 12 years but with severe medical and neurological problems [30,31]. Our patient has a severe phenotype which includes severe global developmental, epileptic seizures, dystonic movements, growth delay, and sensorineural hearing impairment, but without a history of regression or severe deterioration and without lactic acidosis or excretion of methylmalonic acid. Thus, the role of this variant in the child phenotype is currently dubitable. Hearing impairment has been described by a number of authors in connection to SUCLG1 gene mutations and mitochondrial DNA depletion syndrome-9 (Encephalomyopathic Type with Methylmalonic Aciduria) [32,33] and also as uncertain clinical manifestation by others [34,35]. Morava et al. suggested that late-onset hearing loss and absence of ocular involvement are differential diagnostic clues in favor of a defect in SUCLA2 rather than SUCLG1 [36]. Valayannopoulos et al. suggest that statistically significant differences in the clinical features between patients with SUCLA2 or SUCLG1 mutations except from sensorineural hearing loss who had a significantly higher prevalence in SUCLA2 patients [35].

## 4. Conclusions

In the first case two heterozygous variants in the TWNK gene, one likely pathogenic and another of uncertain clinical significance, were detected. This gene is associated with autosomal recessive mitochondrial DNA depletion syndrome type 7 (hepatocerebral type), a severe condition which includes as main clinical features hypotonia, ataxia, ophthalmoplegia, hearing loss, seizures, and sensory axonal neuropathy; these features were present also in our patient. We considered that these two variants are responsible both for hearing loss and neurological problems.

In the second case heterozygous variants of uncertain significance were identified in two different genes: PACS2 and SYT2. Mutations in PACS2 gene were reported in association with autosomal dominant early infantile epileptic encephalopathy, a clinical picture presented also in our patient, whereas pathogenic variants of the SYT2 gene were associated with congenital presynaptic myasthenic syndrome type 7 (not present in our patient). The role of these two gene in the hearing impairment has to be further evaluted.

In the third case a homozygous variant of uncertain significance in SUCLG1 gene was identified; pathogenic variants of this gene were reported in mitochondrial DNA depletion syndrome 9 characterized by severe global developmental delay, hypotonia, hearing impairment, feeding difficulties, hypoglycemia, lactic acidosis, respiratory insufficiency. Although SUCLG1 gene has been reported in association with deafness, the precise role of this variant in our patient phenotype could not be established. Our cases bring new insights in some rare genetic syndromes. Although the role of TWNK gene in hearing impairment is clear and accordingly reflected in published literature as well as in the present article, for the presented gene variants, a correlation to hearing problems could not yet be established and requires more scientific data. We consider that further studies are necessary for a better understanding of the role of these variants.

We should also underline the unpredictable evolution of such cases, with one of the children passing away at the age of 2 years due to numerous complications, whilst the other two children received cochlear implantation with good functional results and reached the ages of 4 and 11 years respectively.

## Data Availability

The genetic testing for all three reported cases has been ordered by the parents and accomplished in private genetic laboratories abroad. Thus, the raw data regarding sequencing was never available for the clinical practitioners that published this work.
